# A survey of physical methods for studying nuclear mechanics and mechanobiology

**DOI:** 10.1063/5.0068126

**Published:** 2021-11-18

**Authors:** Chad M. Hobson, Michael R. Falvo, Richard Superfine

**Affiliations:** 1 Advanced Imaging Center, Janelia Research Campus, Howard Hughes Medical Institute, Ashburn, Virginia 20147, USA; 2 Department of Physics and Astronomy, The University of North Carolina at Chapel Hill, Chapel Hill, North Carolina 27599, USA; 3 Department of Applied Physical Science, The University of North Carolina at Chapel Hill, Chapel Hill, North Carolina 27599, USA

## Abstract

It is increasingly appreciated that the cell nucleus is not only a home for DNA but also a complex material that resists physical deformations and dynamically responds to external mechanical cues. The molecules that confer mechanical properties to nuclei certainly contribute to laminopathies and possibly contribute to cellular mechanotransduction and physical processes in cancer such as metastasis. Studying nuclear mechanics and the downstream biochemical consequences or their modulation requires a suite of complex assays for applying, measuring, and visualizing mechanical forces across diverse length, time, and force scales. Here, we review the current methods in nuclear mechanics and mechanobiology, placing specific emphasis on each of their unique advantages and limitations. Furthermore, we explore important considerations in selecting a new methodology as are demonstrated by recent examples from the literature. We conclude by providing an outlook on the development of new methods and the judicious use of the current techniques for continued exploration into the role of nuclear mechanobiology.

## INTRODUCTION

For generations, it has been known that the cell nucleus houses an organism's DNA and acts as an effective regulator of cellular fate and function.^1,2^ While its biochemical roles are well-established, studies in recent years have increasingly uncovered the importance of the physical nature of the nucleus.^3–5^ Physical processes such as tissue morphogenesis and cancer metastasis can induce significant deformations to nuclei.^6,7^ These deformations are drastic enough to induce DNA damage and even rupture nuclei.^8–14^ Therefore, a growing number of studies have sought to characterize the mechanical properties of nuclei, demonstrating that they are predominantly dictated by chromatin and the nuclear lamina as well as their connections within themselves and to the cytoskeleton.^3,15–21^ Interestingly, alterations in these nuclear constituents and their subsequent material properties have been linked with numerous disease states, including various cancers and laminopathies such as Emery-Dreifuss muscular dystrophy and Hutchinson-Gilford Progeria Syndrome.^22–33^ This is consistent with early observations of compromised nuclear morphology in disease, which implicates nuclear mechanics as a biomarker for disease states.^34–37^ The physical nature of the nucleus, however, is equally relevant to healthy function. Previous observations have shown cells convert mechanical cues into biochemical signals by a process known as mechanotransduction.^38,39^ The same holds true within the nucleus, as it is increasingly evident that nuclei can transduce mechanical forces to alter transcriptional activity and cell function.^40–42^ Studying nuclear deformation, altered nuclear mechanics in disease, and nuclear mechanotransduction, however, necessitates a diverse range of methodologies.

The scope of methods for studying the mechanical properties of nuclei and downstream consequences thereof can be daunting. These techniques span orders of magnitude both in the length scales of their deformations (nanometers to micrometers) and magnitudes of the accompanying forces (picoNewtons to nanoNewtons). Furthermore, there exists a wide variety of the mechanism of perturbation, the direction of force application, specificity of the resulting deformation, and the assay readout. Proper selection of a method and an understanding of its limitations and advantages are paramount for drawing reasonable and accurate conclusions from a mechanobiological study. In this review, we begin by surveying the current methods in nuclear mechanobiology, contextualized by their advantages and limitations as well as how their use has specifically informed our understanding of the cell nucleus. Building on this knowledge, we next discuss some of the most important considerations in the selection of a method and subsequent interpretation of the resulting data. Finally, we conclude this review with an outlook on the present methodologies and how further development and complementary use are necessary as the field continues to grow.

## METHODS IN NUCLEAR MECHANOBIOLOGY

Nuclear mechanobiology is a beautifully complex area of research that necessitates assays for studying both the material properties of nuclei themselves as well as the downstream consequences of nuclear deformation and altered nuclear mechanics. Adding to this complexity, many of the associated phenomena occur on different scales of length, force, and time. For example, beating cardiomyocytes induce small nuclear deformations periodically on the time scale of single seconds.^43,44^ Conversely, cell migration through narrow pores during cancer metastasis causes drastic nuclear deformation that persists over the course of hours.^7,45,46^ These examples are of global deformations to nuclei, but more localized deformations are also physiologically relevant as in the case of microtubule-induced invaginations and fluctuations in the nuclear envelope.^47–49^ Consequently, no one methodology is ideally suited for answering all biomechanical questions as the geometry, force magnitude, and time scale must match that of the physiological process being studied. It is, therefore, important to understand the unique strengths and weaknesses of these techniques and how they can complement each other to ameliorate the limitations of a single assay. Here, we review the most predominantly used techniques for measuring both the material properties of cell nuclei and the downstream effects of modulations of nuclear mechanics (Table I) as well as their unique advantages and limitations (Table II). Before beginning this discussion, however, it is prudent to describe some foundational principles of mechanics and materials.

**TABLE I.** A summary of methods for studying nuclear mechanobiology. Methods within a given class are group by shading.

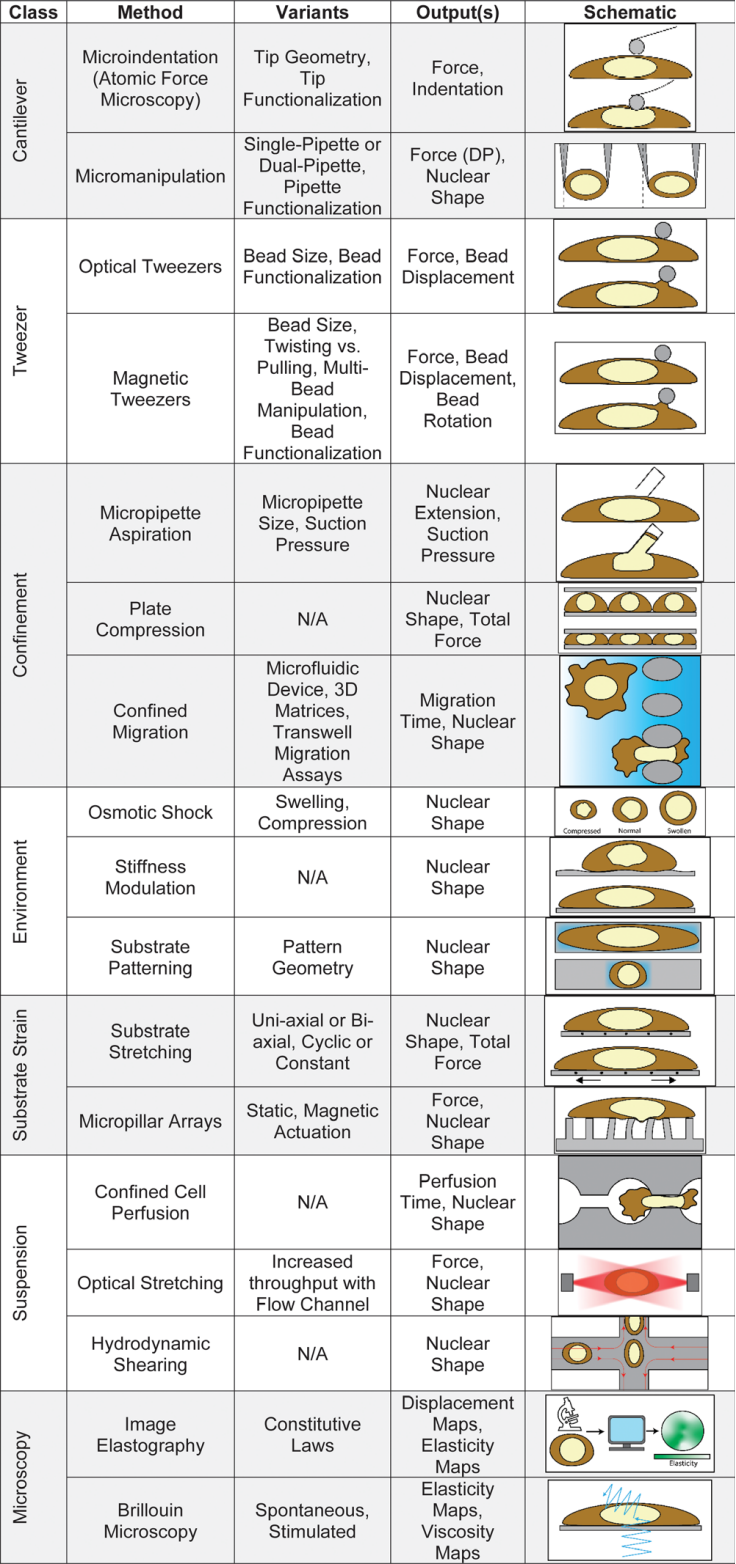

**TABLE II.** Selected advantages and limitations of each class of methods.

Method class	Advantages	Limitations
Cantilever	High dynamic range for stress and strain	Single-cell throughput
		Insensitive to single pN-scale forces
	Simultaneous stress and strain measurements	
		Measurement can be conflated when probing nuclei on soft substrates
	Variable strain rates	
	Sensitive to chromatin- and lamin-based mechanics	
	Capable of ligand specific transduction pathway	
Tweezers	Capable of single pN force measurements	Low dynamic range for stress and strain
	Simultaneous stress and strain measurements	Single-cell throughput (unless parallelized with permanent magnet)
	Highly localized stress application	
	Variable stress profiles (twisting vs extension/compression)	
	Capable of ligand specific transduction pathway	
Confinement	Single- or multi-cell stress application	Limited to physiological cases related to nuclear confinement
	Sensitive to lamin-based nuclear mechanics (MA specifically)	
		Insensitive to chromatin-based mechanics (MA specifically)
	Useful in studying downstream consequences of nuclear deformation	
		Confinement can alter the cytoskeletal organization and cause blebbing.
		Incompatible with isolate
		nuclei (confined migration specifically)
Environmental	Not physically invasive	Not capable of measuring mechanical forces or material properties alone
	Useful in mimicking different physiological conditions	
	Can be coupled with other methods to measure mechanical forces	Necessitates visualization/microscopy when used alone
	High-throughput, multi-cell approach	
Substrate Strain	High dynamic range of strain, frequency, and duration	Limited to lateral strain application
	High-throughput, multi-cell approach	Generally unable to quantify the magnitude of force applied to each cell
		Limited in specificity of strain application
	Useful in studying downstream consequences of mechanical forces	
Suspension	High-throughput, single-cell measurements	Isolating the contribution of the nucleus is nontrivial
	Variable mechanisms of applying stress	
		Limited specificity of strain application
	Substrate does not conflate mechanical measurements	
		Cannot be used in conjunction with monolayers and/or tissue samples
		Rapid timescale of nuclear deformation
Microscopy	Not physically invasive	Subject to all optical aberrations associated with fluorescence microscopy
	Capable of measuring material properties of nuclei	
	Can be used to measure nuclear mechanics *in vivo*	Current debate over the role of water content in measuring elasticity
	Can be coupled with external devices to apply specific strains	
		Necessitates fluorescence microscopy, which can in turn damage the specimen
	Can be used with various models and layers of complexity	

### An introduction to material properties

**FIG. 1.**
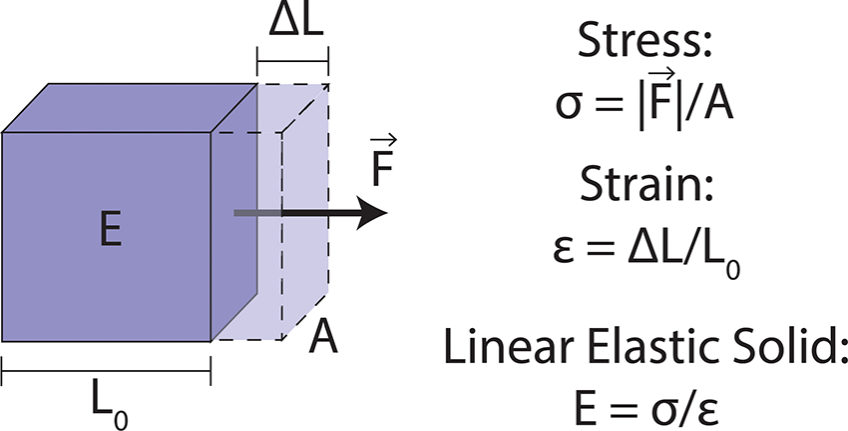
Schematic diagram of a linearly elastic solid. A force ( $\vec{F}$) is applied to the area ( A) of one face of a linearly elastic solid. This stress ( σ) in turn causes a change in length ( ΔL) relative to the initial length of the solid ( $L_{0}$), also known as a strain ( ϵ). The ratio of stress to strain yields the Young's modulus of the elastic solid ( E), a measure of intensive stiffness.

In examining the basics of materials and mechanics, let us consider an elastic solid (Fig. 1). An elastic solid is the simplest material model as it is independent of timescales and rates of deformation. Mechanical processes fundamentally involve forces ( F) and displacement of material ( ΔL). All techniques that are used to probe the mechanical properties or mechanical response of the nucleus require the ability to measure and/or apply one or both fundamental parameters. In material mechanics, stress refers to a force per unit area ( σ=F/A), while strain refers to the fractional change in the length of an object or sub-element of that object ( $\epsilon =\Delta L/L_{0}$). Stress and strain are convenient parameters as they normalize out the scale of the problem and provide, in principle, the intensive mechanical properties of a material. In the case of a linear elastic solid, the stress–strain relation is governed by the Young's modulus ( E=σ/ϵ), which is a measure of the intensive stiffness of a material and is expressed in units of Pa (N/m^2^). It is crucial to note, however, that here we are presenting a simplified version of full elastic theory. When an elastic solid is subjected to a normal stress as shown in Fig. 1, strains are induced along all three axes with magnitudes dictated by the materials compressibility. This compressibility is directly related to a material's Poisson ratio—a common parameter in many models used in analyzing data from force measurement assays. A perfectly incompressible material, for example, has a Poisson ratio of 0.5. For a full discussion of elastic theory, we refer readers to several excellent texts on the topic.^50,51^

Using linear elastic constitutive equations dramatically oversimplifies the complex properties of the cell and nucleus,^52^ however, it is commonly used to provide a basic characterization of cell or nuclear stiffness. The next level of sophistication is to consider the nuclear as a viscoelastic material, whereby both the strain itself and the strain rate affect the resulting stress response. The nonlinearity and viscous nature of the nuclear mechanical properties can be assessed by experiments where the stress and strain are applied over a range of magnitudes and rates. The next level of sophistication is to address the time dependence of nuclear mechanics to and apply viscoelastic constitutive modeling where both the solid-like elastic properties and liquid-like viscous properties can be assessed. For a more thorough discussion on the mechanical properties of nuclei and subsequent analytical and computational modeling, we refer the readers to several review articles.^3,4,17,19,52,53^

### Cantilever-based methods

One of the most popularized means of physically perturbing cell nuclei is a class of methods that we collectively term “cantilever-based” methods. These techniques use flexible cantilevers to stretch or compress individual nuclei while simultaneously monitoring the sample deformation and bending of the cantilever itself. Calibration of the stiffness of the cantilever coupled with precise monitoring of its deflection provides a measure of the force being applied to a nucleus. This general method has been experimentally realized in two primary ways: microindentation^54^ and micromanipulation.^55^

Microindentation comes in several variants of which the most popularized for mechanically probing both isolated and intact nuclei is atomic force microscopy (AFM).^56–72^ An microindentation device functions by gently lowering a flexible cantilever toward a cell or nucleus with one end of the cantilever fixed to a piezo and the other free to deflect upon contact with the sample. In an AFM, a superluminescent diode is reflected off the back of the cantilever onto a quadrant photodiode, enabling quantification of angstrom-scale cantilever deflections and, thus, forces ranging from tens of pN to hundreds of nN. Simultaneously, the indentation of the sample is monitored by measuring the motion of the fixed end of the cantilever, thus enabling quantification of nuclear elasticity. This strategy has been used to elucidate how nuclear stiffness changes in response to, for example, chromatin compaction states,^65^ DNA damage,^72^ and altered lamin levels.^61^ A unique degree of freedom with AFM is the shape of the tip at the free end of the cantilever. Sharp tips have proven useful for studying nuclear rupture,^71^ quantifying more local material properties of nuclei,^65^ or even puncturing the cell membrane to probe the nucleus alone *in situ*.^66,67,73^ Interestingly, AFM does not necessitate the visualization of the sample deformation to quantify effective material properties. However, the addition of fluorescence microscopy to AFM experiments introduces detailed strain mapping that enables further insights. For example, coupling of confocal microscopy with AFM allowed investigators to show that nuclei undergo anisotropic deformation under compression, suggesting nuclear stiffness is different along the major and minor axes.^74^ Furthermore, coupling of side-view light-sheet fluorescence microscopy (LSFM) has proven useful for directly imaging the plane of applied force,^63,75^ enabling investigators to show local nuclear curvature is predominantly dictated by chromatin compaction.^64^ While AFM can apply forces in both the normal and lateral directions, it is predominantly used to apply compression from above, which fails to capture all physiological scenarios in which a nucleus would experience external forces. Furthermore, this direction of perturbation is known to also perturb the underlying substrate when considering cells plated on deformable gels, which can serve to conflate the mechanical measurement.^76^

To accommodate physical perturbations in other directions, investigators have turned to micromanipulation. In brief, a micropipette is physiochemically attached the sample and incrementally moved to induce a lateral strain. This method has been applied to stretch cells,^22,77–82^ isolated nuclei,^14,83–86^ or even individual chromosomes and chromatin fibers,^55,87–89^ where the technique was first developed. This can be done in either a single- or dual-pipette configuration. Single-pipette micromanipulation enables lateral deformations of nuclei with simultaneous monitoring of nuclear shape through fluorescence microscopy. This method has been used to show, for example, the role of vimentin intermediate filaments in protecting nuclear shape change.^79^ The addition of the second pipette on the opposite side of the original pipette, when calibrated for its characteristic stiffness, provides quantification of deflection and subsequently the force applied to the nucleus.^55,83^ This unique method has been leveraged to specifically show how chromatin (eu-/heterochromatin) and lamin A/C separately dictated the nuclear force response at small and large extensions respectively.^83,84^ Coincidently, this was recently shown to also hold true in AFM-based compression.^64^ Both AFM and micromanipulation excel in studies of nuclear mechanobiology at the single-cell level with a high dynamic range of stress and strain as well as simultaneous measurement of force and nuclear deformation; however, alternative approaches are necessary for smaller force scales and localized perturbations, as well as measuring nuclear mechanics *in vivo*.

### Tweezer methods

To apply and measure minute and localized mechanical forces, many investigators opt for “tweezer methods,” either optical or magnetic. Optical tweezers can achieve pN-level force scales with targeted localization through optical manipulation of micrometer-scale beads.^90–92^ After physiochemically linking beads to a cell or nucleus, a focused beam of light is used to “trap” and subsequently translate the bead. Displacement of the bead from the center of the optical trap is used to quantify forces down to single picoNewtons. The high sensitivity of optical tweezers has been foundational in establishing the role of chromatin in nuclear mechanics. For example, experiments with optical tweezers have shown that nuclei increasingly soften upon continued enzymatic chromatin decondensation.^93^ More specifically, it was later shown with that chromatin tethering to the nuclear envelope was critical in stiffness regulation.^21^ The relatively small-scale stresses and strains that optical manipulation methods provide have unearthed new territory in nuclear biomechanics; however, they are not the only means of applying such local and minute perturbations.

Akin to optical tweezers, manipulation of magnetic particles with precisely controlled magnetic fields—known as magnetic tweezers—enables pN to nN level force measurement and application on individual nuclei.^94–96^ Magnetic manipulation as opposed to optical manipulation, however, enables additional variations in particle actuation. In addition to 2D translation, 3D magnetic tweezer assays have been developed to precisely displace beads in all spatial directions.^97^ Additionally, magnetic fields can rotate magnetic particles, thus providing an alternative stress profile.^38^ While this is typically used as a single-cell, low-throughput technique, the use of magnets to simultaneously apply stress to numerous cells has proved useful for studying alterations in protein expression in response to external stress.^98^ Magnetic tweezers have recently been used to show polarity in nuclear mechanical properties^99^ as well as how the Linker of Nucleoskeleton and Cytoskeleton (LINC) complex mediates nuclear stiffening in response to repeated force application and that the nucleus is an active, adaptive material.^98^ In addition to accessing very low force magnitudes, these methods enable localized perturbations to nuclei similar to those attainable by cantilever-based methods. Bead and cantilever methods also share the ability to study the force transmission pathway from cell exterior to the nucleus through the control of the functionalization of the probe, thereby controlling which cell surface receptor is transmitting the force. This is especially powerful for studying force transduction from the cell surface to the chromatin,^38,63,100^ which has been further used to show chromatin strain leads to transcription upregulation^101^ and depends upon histone methylation^102^ as well as the orientation of actin stress fibers.^103^ Bead methods have unique advantages when applied within the cell or nucleus. Recently, magnetic manipulation has been pushed even further to study *in situ* interphase chromatin mechanics. By tethering magnetic nanoparticles to genomic repeats, the investigators were able to magnetically perturb an individual genomic locus.^104^ This new method could prove complementary to well-established tweezer methods for studying chromatin mechanics.^105^ Out of all the methods described in this review, tweezer-based approaches are exceptionally well-suited for studying local, minute stress and strain and the resulting downstream consequences. There exist several physiological scenarios, however, where much larger forces are relevant.

### Confinement-based methods

Nuclei undergo significant compression and deformation in a variety of physiological processes, ranging from metastasis to tissue morphogenesis.^6,7^ “Confinement-based” techniques are well-suited to mimic such scenarios as they provide the required global compressions. Several variants of confinement-based methods have been developed over recent years, but the underlying common theme is a significant compression of a nucleus between rigid surfaces. The variations come both in how nuclei enter these confinements, the geometry of the confinements themselves, and the overall throughputs of the assays.

A popular confinement-based method in nuclear mechanobiology is micropipette aspiration (MA). In an MA experiment, a micropipette with a prescribed opening (typically several micrometers in diameter) and a controlled internal pressure is used to aspirate a whole cell or more usually a local region of a nucleus into the pipette. This subsequently induces nuclear strains on the order of 50%–200%. Simultaneously, fluorescence or bright field microscopy is used to track the movement of the sample into the micropipette.^106,107^ Together, this combined aspiration and visualization have been instrumental for two primary functions: evaluating viscoelastic properties of nuclear lamins and studying downstream effects of physical compression. Regarding the former, this is performed traditionally by fitting a mechanical model to the time series of the nuclear length within the pipette.^108^ Such analysis was foundational in establishing both that nuclei are stiffer than the surround cell body^109^ as well as the role of the nuclear lamina and surrounding proteins in nuclear mechanics and its subsequent implication in disease states.^57,110–115^ In addition, later work used MA to tease out that the ratio of A- and B-type lamins scales with nuclear stiffness.^116,117^ However, MA is useful beyond just studying lamin-based nuclear mechanics in that it enables visualization of a cell's response to nuclear confinement. For example, fluorescence imaging of both histones and various mobile nuclear proteins showed that during aspiration of nuclei into constrictions segregated the mobile proteins from the chromatin.^118^ Further modeling has indicated that this may be a mechanism by which nuclei incur DNA damage under large deformations.^119^ Micropipette aspiration excels at probing the nuclear lamina as well as the effects of sustained nuclear confinement. Despite recent advances,^120,121^ MA remains primarily a single-cell assay.

Potentially, the simplest realization of confining a nucleus is through parallel plate compression.^122,123^ While this method can assess material properties of individual nuclei,^124^ the true benefit of this method is that confinement can be applied simultaneously to an entire cell monolayer, providing dramatical parallelization of the compressive measurement. However, the parallelization subsequently limits the ability to precisely measure the forces or stresses upon an individual nucleus. The primary readout is then typically from correlative fluorescence microscopy. This simple—yet powerful—assay is invaluable for studies of nuclear mechanotransduction. Namely, investigators have shown how nuclear confinement sufficiently alters transcriptional activity,^125,126^ and that the resulting deformation is sufficient to induce DNA damage independent of nuclear rupture in certain cell lines.^12^ Furthermore, a recent pair of studies used similar confinement assays to show how nuclear compression beyond a specific threshold leads to tension in the nuclear envelope and significantly increases rapid, stable, and reversible actomyosin contractility.^127,128^ The minimal complexity, high throughput, and rapid biological readouts of this assay have even further potential in a clinical setting for discriminating between healthy and cancerous cells.^129^

As cells migrate throughout the body, particularly in the case of metastasis, they often encounter narrow interstitial spaces which force the nucleus to undergo drastic deformation.^130^ In studying such processes, investigators have developed a breadth of devices to replicate such extracellular environments. As has been previously reviewed,^131^ three primary *in vitro* variants have surfaced as the most prominent: microfluidic devices,^132,133^ transwell migration assays (or Boyden migration assays),^134,135^ and 3D collagen matrices.^136,137^ Though slightly different in their specific approaches, each assay aims to emulate the *in vivo* 2–20 *μ*m constriction nuclei experience during migration.^136^ Unlike plate compression or MA experiments, these migration assays leverage chemoattractant gradients to spur directed cell migration through the confinements. Doing so has provided compelling evidence for the relevance of the nuclear mechanical properties in migration and metastasis. It was first shown how the nucleus, being the stiffest and largest subcellular component, provides the rate-limiting step during confined migration.^45,116^ More specifically, it was highlighted that the nuclear lamina is the dominant mechanical component for these processes, and that softening of the lamina promotes migration through confinements.^45,46,116,138–140^ Furthermore, it has been observed that the LINC complex is essential for pulling nuclei through constrictions,^141,142^ but doing so can inherently lead to plastic damage to nuclear shape.^46,133,143,144^ Building on these works, it was later demonstrated that confinement was sufficient to induce nuclear rupture and subsequent DNA damage due to cytoplasmic nucleases entering the nuclear interior.^9–11,118,145^ DNA damage is not the only downstream effect that has been visualized with these devices. The role of chromatin in migration is increasingly being appreciated,^146^ and it has recently been observed that despite the need from chromatin condensation upon induction of migration,^147^ migration through narrow spaces leads to chromatin decompaction and nuclear softening to facilitate passage.^148–150^ A further important consideration is that confined migration assays are incompatible with isolated nuclei, unlike all other methods mentioned here. Therefore, these assays may struggle more so to isolate nuclear contributions to the cellular response. Though we have opted to include statements on compatibility with intact vs isolated nuclei, a full discussion of comparative mechanics is beyond the given scope of this review. Compression is also not the only relevant stress a nucleus may feel during, for example, tissue morphogenesis. It is, therefore, prudent to next discuss methods of modulating the surrounding environment to induce alternative deformations.

### Environmental modulations

The next means of studying nuclear mechanobiology comes through modulation of the environment in which cells and tissues are grown. This can practically be achieved through modulation of the surrounding media as well as the substrate itself. Beginning with the former, the predominant means of modulating the environment is through changes in the media osmolarity, known as osmotic shock. By altering the concentration of solutes in the media, a pressure gradient across the cell membrane and nuclear membrane is induced. This causes a flux of water through the membranes, thus compressing or swelling the cell and nucleus, depending on whether the concentration of solutes in the media is raised or lowered respectively. This provides a noninvasive means of modulating nuclear morphology, which has proved to be a useful method for studying both downstream consequences of nuclear deformation as well as the mechanical properties of nuclei.^151^ Osmotic shock is often coupled with volumetric imaging of nuclei to study the relationship between pressure and volume.^152–155^ This has shown, for example, that nuclear volume scales nonlinearly with osmolarity, implying an additional force necessary to stretch the nuclear envelope.^152^ Alternatively, osmotic shock of nuclei has been shown to alter chromatin condensation and nucleocytoplasmic transport, potentially signifying a mechanism of nuclear mechanotransduction.^156–158^ Furthermore, using altered osmotic conditions to swell or shrink nuclei has proven useful when combined with other methods such as micropipette aspiration for showing the consequences of nuclear volume change on its mechanical properties,^111^ potentially pointing toward the importance of considering nuclear compressibility and the Poisson ratio in mechanical measurements.

Environmental modulations can also be made to the substrate upon which cells and tissues are grown. Substrate alterations can either be to the mechanical properties of the substrate or the substrate's topography. Evidence that cells can biochemically respond to mechanical changes in the surrounding environment has been well documented.^39,159^ It has since been shown that similar changes to matrix elasticity can, in turn, alter nuclear shape, lamin A transcription, and tension in the nuclear lamina.^117,160^ However, it is crucial to consider that a soft gel may also be deformed when used in concert with a physical perturbation of a cell nucleus, potentially conflating the resulting measurement.^76^ Alternatively, one can control areas of the substrate where a cell may spread or migrate. This is practically achieved through micropatterning of adhesion molecules such as fibronectin or physically manufacturing substrates with patterned topography. Though simply altering the surface topography or patterning provides no measure of force or strain, this high-throughput method is well suited for studying the relationship between cellular and nuclear shape and their downstream consequences. Plating cells on small, circular patterns as opposed to wider rectangular patterns revealed that cell spreading and the development of perinuclear actin stress fibers flatten nuclei, decrease chromatin condensation, induce tension in the nuclear lamina, and minimize nuclear shape fluctuations.^125,161,162^ In the context of migration, substrate patterns that transition from thin lines to wide rectangles have informed how nuclear shape mimics that of the surrounding cell body.^163,164^ Additionally, substrates with sinusoidal axial curvature have been used to show the cells can sense local curvature and position their nuclei near topographical minima.^165^ Though these methods themselves do not measure mechanical forces and require microscopy to visualize the implications of environmental modulations, they excel at mimicking a range of physiological conditions and can easily be coupled with many of the other methods described in this review.

### Substrate strain

Physical stretching of elastic (or flexible) substrates is one of the oldest methods for investigating the impact of mechanical forces on the cell nucleus.^166,167^ The principle is straightforward in that cell monolayers are cultured on deformable, elastic substrates that are mechanically linked to actuators that induce strain throughout the surface.^168–170^ A remarkably high-throughput assay, substrate strain devices are exceptional for quantifying biochemical responses to nuclear strain across a range of strains (3%–50%), frequencies (static—∼1 Hz), and durations (minutes–days). Specifically, it has been used for highlighting how the state of chromatin compaction may be altered as a result of prolonged strain within the nucleus.^13,171–175^ While this particular method is unable to elucidate material properties of the nucleus, monitoring the magnitude of the nuclear shape change due to a given substrate strain is an effective method for determining which cellular structures are responsible for minimizing nuclear deformation. For example, by quantifying changes in nuclear strain due to substrate stretching, investigators were able to tease out that A-type lamins and not B-type lamins are responsible, in part, for the mechanical response of the nucleus.^176,177^ It was similarly shown that the mechanical integrity of the nucleus is compromised in muscle-phenotype laminopathies.^22^ Finally, it has been more recently documented that chromatin and the apical stress fibers can act to resist nuclear strain^13,178^ along with the nuclear lamina.

Substrates populated with micropillar arrays have also been used for investigating nuclear mechanics. Such arrays have been a common approach for studying forces associated with cell motility;^179^ recent technical advancements have built on this approach to enable localized magnetic actuation of micropillars. By partially filling micropillars with magnetic material during the fabrication process, investigators use micropillars to both apply and measure cellular scale forces.^180^ This technique was more recently applied to study the mechanobiology of the nucleus, showing specifically that periodic pinching via magnetic micropillars led to shuttling of megakaryoblastic acute leukemia factor-1 (MKL), a transcription cofactor, out of the nucleus and into the cytoplasm.^181^ To date, this assay has been underutilized relative to many of the other methods mentioned in this review. However, it is poised to be extremely beneficial as it allows for precise, subcellular, calibrated force application to live cells. More commonly, investigators have used rigid micropillars as a means of inducing nuclear deformation.^182–185^

### Suspension methods

While most studies of the mechanics of the nucleus are performed on cells adhered to a substrate, there are a set of methods that measure the mechanical properties of cells in suspension. These methods are advantageous for two reasons: (i) they offer the opportunity for high throughput analysis similar in methodology to fluorescence activated cell sorting (FACS), and (ii) they remove the complexity of the substrate altering the cell and nuclear mechanical properties. In these experiments, the suspended cells are spatially flow-aligned in a microfluidic channel then controllably passed across a sensing zone. Three methods of this kind apply stress through physical constrictions, optical fields, or hydrodynamic forces.

Suspended cell measurements based on physical constrictions have been employed to study cell mechanical properties in several reports.^166,186–192^ Quantification of the time it takes a cell to passage through these confinements provides a metric for mechanical resistance to deformation. Though not extensively used, these assays have been leveraged to demonstrate how increased levels of lamin A/C slows cell passage through narrow pores.^45,139^ Most interesting is when these methods are automated and integrated with electronic sensing to simplify the instrumentation and allow integration with other methods. This is the case for visco-node-pore sensing (visco-NPS), which integrates a microfluidic channel of cellular dimensions with integrated electrodes to measure the channel conductance.^193,194^ As a cell moves through the channel, it encounters sinusoidal constrictions that compress the cell—and dramatically reduce the ionic current through the channel. The time dependence of the single cell translation down the channel is then measured by the alteration of the electrical current sensed through the fluid using the electrodes that are placed at the opposite ends of the channel. The initial size of the free cell is measured electrically based on the Coulter principle, so the entire measurement requires no imaging. Together, this assay can measure approximately 10–50 cells/min while applying strains on the order of 30%–50%. To assess the mechanical resistance of the nucleus specifically, the cells were treated with Latrunculin B to depolymerize the actin cytoskeleton.^193,194^

Constriction based methods involve the contact of the chamber walls with the cell under study. However, forces can be applied to the cells through hydrodynamic forces that occur under shear and extensional flows.^195,196^ Shear flows occur anytime there is flow near a boundary as the no-slip boundary condition ensures there will be a gradient of fluid velocity from the wall to the channel center. In a channel of suitably small dimensions, this shear flow field is sufficient to deform the cell. While the geometry may appear to be the same as the constriction-based assays, the channel dimensions remain larger than the cell, so the cell never touches the wall. This is also known as real time or shear deformation cytometry.^197^ Another version of hydrodynamic forces involves a cross flow geometry that generates an extensional force on cells, termed extensional flow deformation cytometry (eDV).^197^ To generate significant deformations, the flow rates need to be high which confers the advantage of high throughput cell measurements. However, this also means that the method is restricted to very high strain rates, on the scale of kHz. Impressively, extensional flow cytometry has been used to quantify 15 biophysical parameters at a rate >1000 cells/s.^198^ This parameterization was sensitive to the effects of lamin knockdowns on the overall cell deformation properties. A recent comparison of the microfluidic high throughput methods highlights the issue with the suitability of methods for cell nuclear studies.^198^ A common set of cells and interventions were studied using constriction deformation cytometry (cDC), extensional flow deformation cytometry (eDV) and shear deformation cytometry (sDC). While all three methods had similar responses to osmotic shock treatments of the cells, only the cDC and sDC were sensitive to a cytoplasmic intervention. In general, these methods do not measure the deformation of the nucleus explicitly and so can only infer the nuclear mechanical properties through the interpretations of cellular interventions that disrupt the cytoplasm or nucleoplasm. The camera-based versions of these assays can, in principle, be extended to use fluorescent markers or bright field image analysis to assess geometrical parameters of the nucleus from which mechanical properties might be deduced.

In a similar perfusion assay for suspended cells, mechanical forces can also be applied by optical means, specifically known as the optical stretcher.^199–202^ This technique uses two nonfocused, oppositely directed beams aimed at a cell or nucleus, which together generate a net force along the beam axis.^199–201^ This remarkable method has the capability to apply forces on the scale of single pNs, reaching sensitivity well-below that of cantilever-based methods. The combination of the optical stretcher with microfluidic cell delivery devices dramatically increases throughput despite the limitation to serial single-cell measurements.^203^ Optical stretching was used to provide early evidence that decompaction of interphase chromatin leads to overall softening of cell nuclei.^204^ This device, however, similar to the hydrodynamic methods discussed above, induces a global strain across the entire nucleus, which limits spatial specificity. Furthermore, the benefit of exceptionally high throughput comes at the cost of rapid nuclear deformations, which limit the ability to probe solely the elastic contribution of the nuclear force response.

### Microscopy-based methods

In studying nuclear mechanobiology *in vivo* during processes such as tissue morphogenesis, tumorigenesis, or embryogenesis, access to directly probe the nucleus is limited, and it can only be visualized as the physiological processes occur. A final class of methods we note as “passive” or “microscopy-based” can then be leveraged to improve our understanding of the nucleus' role in such contexts. Passive methods can be used to study both viscoelastic properties of nuclei as well as intranuclear transport and dynamics. Regarding the former, development of these methods is an active research area and their implementation is less established. Here, we note two methods with particular promise. The first is a technique known as image-based elastography,^205^ which was recently applied to the problem of nuclear mechanics.^206,207^ In this method, deformation microscopy^208^ is used to generate displacement fields from images of fluorescently labeled histones during nuclear deformation (in this instance, beating of cardiomyocytes). Subsequently, computational analysis assuming a particular material model then backs out the relative elasticity of heterochromatin and euchromatin during the deformation. Though only the ratio of elasticity is available, this is particularly powerful because it can be performed during any *in vivo* process, allowing for new insights into how chromatin mechanics may change during development. Furthermore, the framework is able to be expanded to include more materials, such as the nuclear lamina, in the analysis.^206^

The second method of interest is Brillouin microscopy,^209–211^ which despite its invention approximately 100 years ago has only recently been introduced to biology in the past 15 years.^212^ This image-based technique leverages frequency-shifted scattered light to infer local elasticity and viscosity of biological tissue. Brillouin microscopy is beginning to become more prevalent in nuclear mechanics studies as it has been used to probe local viscoelasticity of nucleoli^213^ as well as validate previous results regarding the mechanical role of lamin A/C and chromatin compaction.^214^ However, despite the positive outlook for Brillouin microscopy as a noninvasive method for studying mechanical properties, there is still debate over precisely what is being measured in these studies.^215,216^ Further passive methods—both well-established [e.g., Fluorescence Recovery After Photobleaching (FRAP), Fluorescence Correlation Spectroscopy (FCS)] and recently developed (e.g., displacement correlation spectroscopy)—are inherently focused on intranuclear diffusion and dynamics, which is beyond the scope of this review.^217–232^ The notion of measuring mechanical properties directly through imaging is a highly active research area, and the continued development of these methods has the potential to shape how nuclear mechanobiology is studied in a more physiologically relevant context.

## CONSIDERATIONS IN CHOOSING A METHOD

No single method can serve the purposes of every study, hence, the development of the myriad of techniques described above. Every technique has strengths and limitations that make it suitable for a subset of modern nuclear mechanobiology insights; however, there is often overlap in that several assay may be well suited for investigating a specific problem. Here, we describe some of the overarching considerations in choosing a technique and highlight recent works as relevant examples to promote effective utilization of the strengths of each modality.

### Throughput vs specificity

**FIG. 2.**
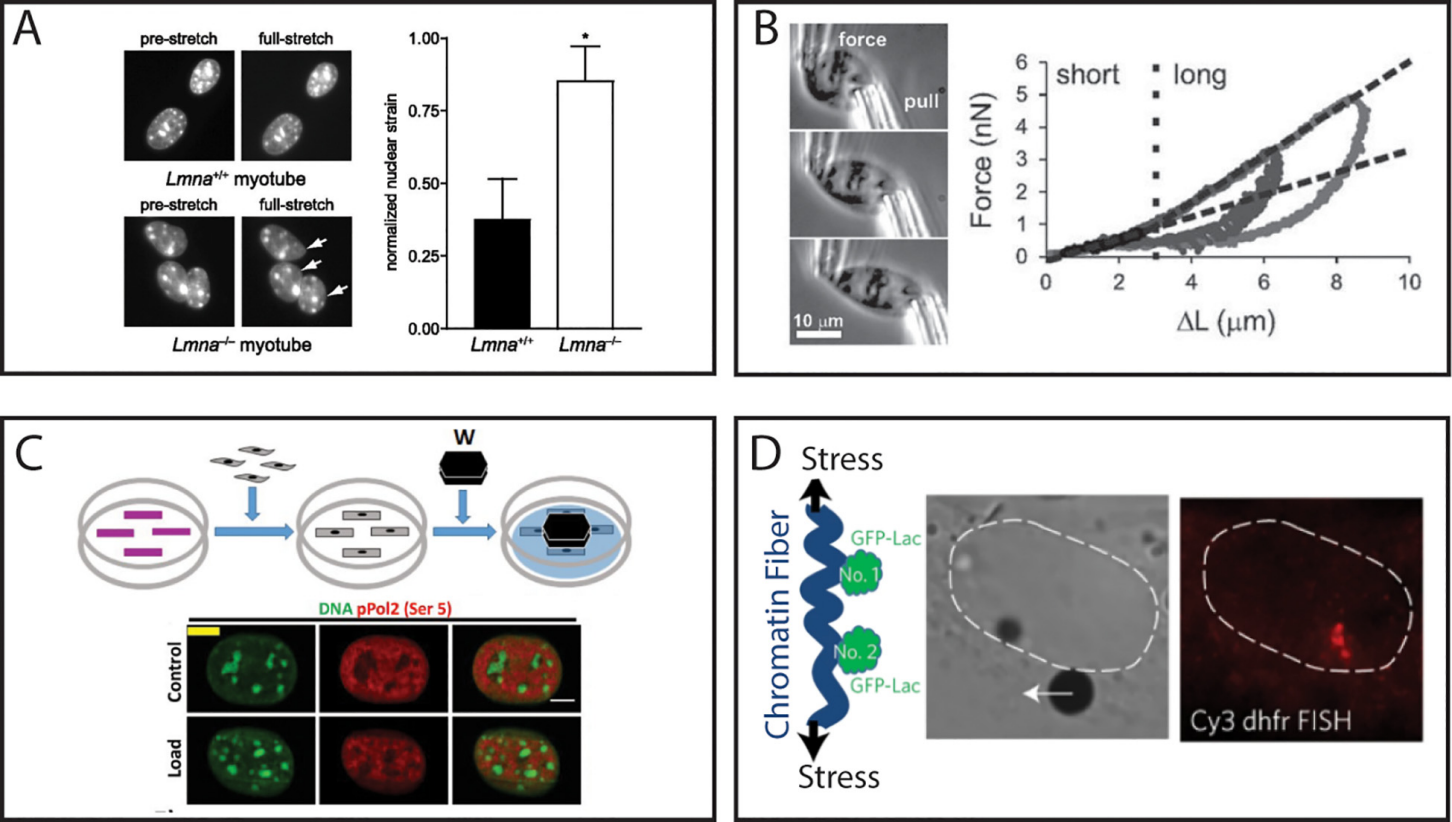
Considerations in specificity vs throughput. (a) Lammerding *et al.* used a substrate strain assay to show how A-type lamins and not B-type lamins are relevant for nuclear mechanics. The bar graph shows nuclear strain for LMNA*+/+* and LMNA*−/−* nuclei, highlighting the role of lamin A/C in nuclear strain response. (b) Stephens *et al.* used dual-pipette micromanipulation to show a nonlinear nuclear force response to stretching, dictated by chromatin and lamins at short and long extensions, respectively. The plot shows nuclear force response as a function of strain, highlighting two regimes of nuclear deformation. (c) Damodaran *et al.* used a plate compression assay to show that global nuclear compression increases chromatin compaction and represses transcriptional activity. (d) Tajik *et al.* used twisting magnetic bead manipulation to show how local stretching of chromatin leads to transcriptional upregulation at the site of strain. Images from (a) are reproduced with permission from Lammerding *et al.*, J. Biol. Chem. **281**(35), 25768–25780 (2006). Copyright 2006 Authors, licensed under a Creative Commons Attribution (CC BY) license. Images from (b) are reproduced with permission from Stephens *et al.*, Mol. Biol. Cell **28**(14), 1984–1996 (2017). Copyright 2017 Authors, licensed under a Creative Commons Attribution (CC BY) license. Images from (c) are reproduced with permission from Damodaran *et al.*, Mol. Biol. Cell **29**(25), 3039–3051 (2018). Copyright 2018 Authors, licensed under a Creative Commons Attribution (CC BY) license. Images from (d) are reprinted with permission from Tajik *et al.*, Nat. Mater. **15**(12), 1287–1296 (2016). Copyright 2016 Springer Nature Customer Service Center GmbH.

Tradeoffs are inherent to the selection of any method; potentially, the most important is choosing between throughput vs specificity. In this case, specificity is defined as the amount of information garnered from a given experiment. One such example is in the consideration of the need for a calibrated force measurement. On the one hand, the techniques capable of measuring with accuracy the magnitude of force being applied to a nucleus are generally single-cell approaches (AFM, MM, optical and magnetic tweezers, etc.). On the other hand, one may exchange this specificity of quantifying the applied force for the high-throughput nature of other methods (substrate stretching, plate compression, etc.) that simultaneously apply global stresses to multiple cells. This is not to say, one such method is preferable to another, but rather that the selection depends upon the question at hand. As an example, consider the studies of Stephens *et al.*^83^ and Lammerding *et al.*^176^ In the latter work, the investigators used substrate stretching to show that A-type lamins as opposed to B-type lamins are relevant to minimize nuclear strain [Fig. 2(a)].^176^ This experiment did not necessitate a calibrated force measurement as the authors sought to highlight the importance of specific lamin isoforms as opposed to quantify specifically the material properties of the nuclei themselves. By using a substrate stretching approach, the authors were able to investigate numerous cells simultaneously, thus improving sample size and minimizing selection bias. To contrast and complement this work, Stephens *et al.* used a calibrated micromanipulation approach to quantify the force experienced by an individual, isolated nucleus for the entire extent of a physical extension [Fig. 2(b)].^83^ Though lower in throughput, this allowed the investigators to observe a nonlinearity in the force response of the nucleus during stretching, which was then attributed to separate roles of chromatin and lamins at short and long extensions, respectively.^83^ Such conclusions would not have been possible without the calibrated force measurement associated with dual-pipette micromanipulation. These examples highlight how proper assay selection to match the research question at hand allows one to draw on the benefits of a given method to bolster their conclusions.

The trade-off between specificity and throughput is also relevant in other contexts. Consider next the case of how external stress may alter transcriptional activity. In studying such phenomena, the emphasis is less on the mechanical properties and response of the nucleus and more so on the biochemical readout of transcriptional activity. However, considerations of specificity are still highly relevant, though in a slightly different context than the previous examples. For this problem, the specificity is in the precision of strain application. Global strain application methods, such as substrate stretching and plate compression, do not allow the user to specify the precise intranuclear strain, but rather the total strain on the substrate or final distance between plates, respectively. This lack of specificity, however, enables a dramatic increase in throughput as these methods can be used to strain entire cell monolayers simultaneously. The benefits of such an approach are exemplified by Damodaran *et al.*^125^ In studying the relationship between compressive force, chromatin compaction, and transcriptional activity, Damodaran *et al.* used a plate compression assay to simultaneously compressed cells and nuclei cultured on patterned substrates [Fig. 2(c)]. The throughput of this assay allowed them to screen a large sample size across multiple substrate pattern geometries and extract readouts of transcription for broad range of genes. Together, this enabled the investigators to conclusively show that compressive force leads to chromatin compaction and subsequent decrease in transcriptional activity.^125^ However, this global approach did not allow the investigators to precisely map local changes in transcription with local strains. For this, we highlight the work of Tajik *et al.*^101^ To study this exact issue, the investigators employed a twisting magnetic bead assay to locally stretch chromatin as was visualized by tracking individual Green Fluorescent Protein (GFP) foci on a single chromatin strand [Fig. 2(d)]. Coupling this assay with fluorescence *in situ* hybridization (FISH), Tajik *et al.* visualized changes in transcriptional activity at the precise site of strain being monitored. This pinnacle of specificity came at the price of throughput in that it does not feature the same level of parallelization as the previous example. However, the gain in specificity enabled them to display how local stretching of chromatin, in turn, upregulates transcription at the site of strain and intricately depends on the mechanical linkages between integrin receptors and the nuclear interior.^101^ Though both interested in the same overarching topic, these two works show how the specific question determines the relative merit of specificity vs throughput when choosing the appropriate assay.

### The shape and direction of the perturbation

**FIG. 3.**
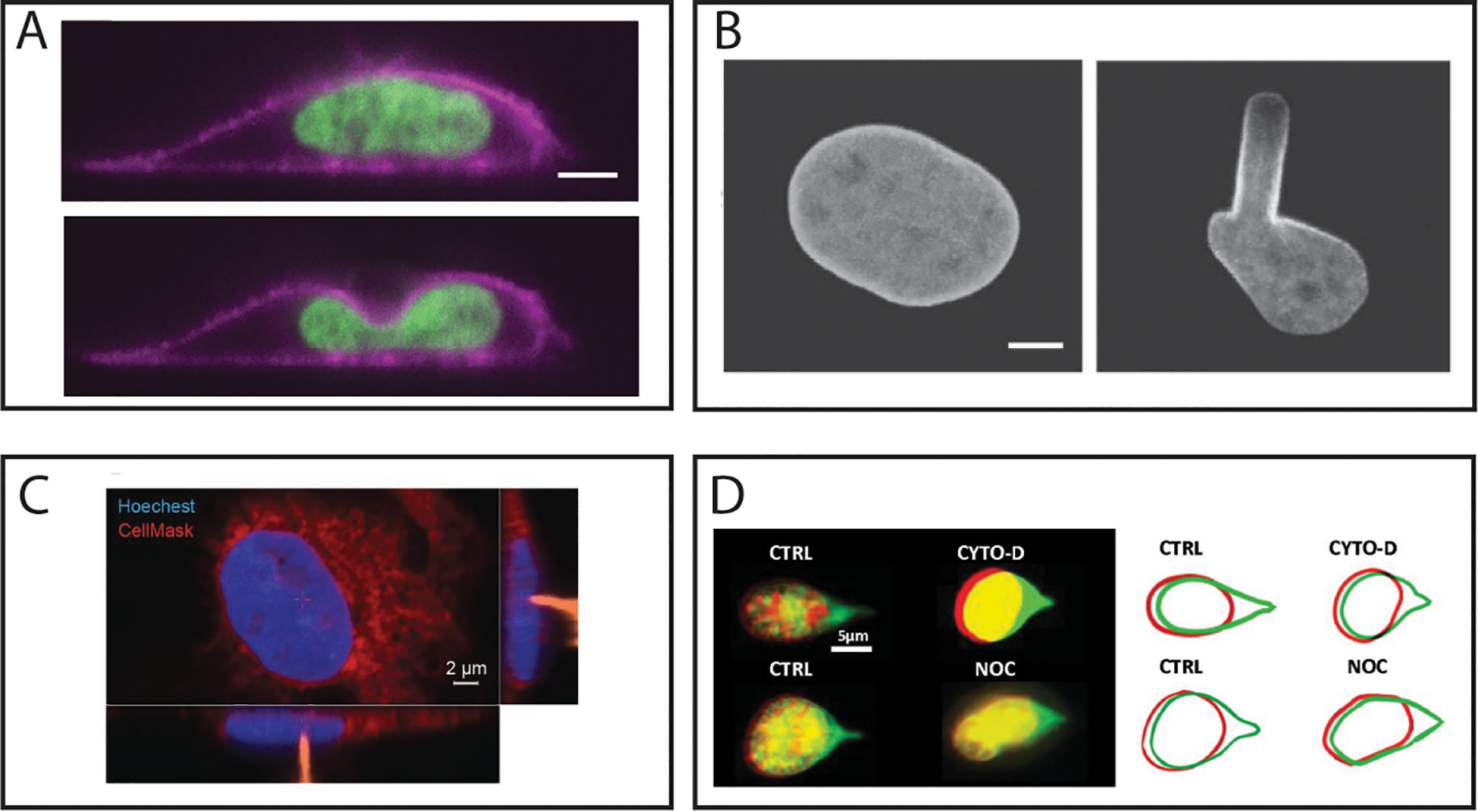
Considerations in the geometry of nuclear perturbation. (a) Side-view images of a nucleus being compressed by an AFM with a 6 *μ*m diameter beaded tip, as visualized in Hobson *et al.* (b) Nuclear deformation during an MA experiment, as visualized in Rowat *et al.* (c) Wang *et al.* used AFM to show inhibition of the actin cytoskeleton reduced the viscoelastic response of the nucleus. Images show the nucleus (blue) being perturbed via a sharp AFM tip (orange) from above. (d) Neelam *et al.* used single-pipette micromanipulation to show inhibition of the actin cytoskeleton did not alter the nuclear response to external force. Red and green images and outlines represent nuclear shape before and after stretching with a micropipette. Images from (a) are reproduced with permission from Hobson *et al.*, Mol. Biol. Cell **31**(16), 1788–1801 (2020). Copyright 2020 Authors, licensed under a Creative Commons Attribution (CC BY) license. Images from (b) are reproduced with permission from Rowat *et al.*, Biophys J. **91**(12), 4649–4664 (2006). Copyright 2006 Elsevier. Images from (c) are republished with permission from Wang *et al.*, J. Cell Sci. **131**(13), jcs209627 (2018). Copyright 2018 The Company of Biologists Ltd., Clearance Center, Inc. Images from (d) are reproduced with permission from Neelam *et al.*, Proc. Natl. Acad. Sci. U. S. A. **112**(18), 5720–5725 (2015). Copyright 2015 National Academy of Sciences.

All nuclear deformations are not created equal. Each of the methods covered in this review perturbs the nucleus is a unique manner. Take, for example, a comparison between AFM and MA. Both would broadly be considered as compressive techniques; in AFM the nucleus is compressed with the flexible cantilever and in MA the nucleus is aspirated into a confining pipette. Despite the broad similarities in nuclear compression, it can be clearly visualized that the shapes of the resulting nuclear deformations differ significantly. Using a 6 *μ*m diameter bead as the tip of the AFM cantilever, the direct side-view imaging performed by Hobson *et al.*^64^ displays how the nucleus seemingly cups around the AFM tip, forming a smooth curvature and local indentation [Fig. 3(a)]. In the case of MA experiments, however, the difference in the deformation is quite stark. As was visualized in Rowat *et al.*,^114^ the aspirated nucleus forms a long protrusion into the pipette with a sharp curvature at the entry point [Fig. 3(b)]. While these differences may appear nuanced and unimportant in the greater context, the implications are not insignificant. By developing nearly identical continuum mechanics models of both AFM indentation and MA that account for the material properties of the inner and outer nuclear membrane, the nuclear lamina, and the chromatin, Vaziri *et al.*^233,234^ demonstrated that the geometry of the deformations for each assay selectively probe different mechanical constituents of the nucleus. Specifically, MA results were particularly sensitive to changes in the material properties of the nuclear envelope and nuclear lamina,^233^ whereas AFM experiments were sensitive to changes in both the material properties of the lamina and chromatin/nucleoplasm.^234^ This implies MA measurements reflect nuclear lamina mechanics, while AFM measurements reflect both the chromatin and lamina mechanics. This potentially explains why early MA experiments concluded that the lamina was the primary mechanical constituent of the nucleus and that chromatin had little role in nuclear deformations,^111^ as well as why recent MA data suggest inhibiting histone deacetylation has no effect on nuclear mechanics.^121^ Similarly, though not well established in the literature at this point, is the notion of changes in volume due to nuclear perturbation. As previously mentioned, materials are not just described by an elastic modulus, but also their compressibility. Some nuclear perturbations may result in strains that cause more, less, or no volume loss due to flux through the nuclear envelope. Careful consideration of this potential volume change must be accounted for when interpreting the material properties of the nucleus, as improper consideration may conflate elasticity and compressibility. This is not to say, one method is preferrable in general, to the other, but rather that one must be interpret their data in the context of their assay, because the geometry of the deformation alone may sufficiently enhance and diminish the contributions of specific nuclear structures.

Equally important to the shape of the deformation is the direction from which the nucleus is perturbed. Nuclei are far from isotropic and respond differently to forces in different directions. To further conflate the issue, some of the surrounding cellular structures are not uniformly distributed nor symmetrically organized around the nucleus. The most prominent example is the actin cytoskeleton, which in well-spread cells forms a series of cables spanning the top of the nucleus.^178,235–237^ Presumably when probing an intact nucleus from above, these apical stress fibers will be engaged in the force response. Likewise, when probing a nucleus from the side, these stress fibers will not contribute to the nuclear force response. When collectively looking at the literature, it appears as though this may be the case. For example, Wang *et al.*^67^ used AFM with a sharp tip to measure the elasticity and viscosity of the nucleus with and without an intact cytoskeleton [Fig. 3(c)]. Not surprisingly, they observed that depolymerization of the actin cytoskeleton via treatment with Cytochalasin D reduced the collective elasticity and viscosity of the nucleus. However, when Neelam *et al.*^79^ used the same cytoskeletal intervention in conjunction with single-pipette MM, they observed no change in the nuclear response compared to control conditions [Fig. 3(d)]. Though these results may initially be viewed as conflicting, consideration of the direction from which the nucleus was probed could provide an explanation of this discrepancy. More symmetric structures such as the perinuclear vimentin cage, however, has been shown to be relevant to the nuclear response to applied for from all directions.^79,238,239^ Investigators must then be judicious both in selection of an assay and interpretation of the results when it pertains to cell nuclear mechanics.

### The importance of length and time scales

The nucleus is a wonderfully complex material, consisting of intricate crosslinked polymers meshes with a semi-permeable membrane and partially fluid-filled interior. Unsurprisingly, material descriptions of the nucleus are nontrivial and the force response is dependent upon the choice of deformation parameters (magnitude, duration, speed, and frequency). For example, the dependency of the nuclear force response on strain magnitude is best demonstrated by the previously described dual-pipette micromanipulation study by Stephens *et al.* first demonstrating that nuclei undergo lamin-based strain stiffening at large extensions.^83^ This was later verified to also be true for nuclear compressions, as was demonstrated by combined AFM and light-sheet fluorescence microscopy.^64^ Interestingly, an increase in nuclear stiffness with increasing strain has also been observed by oscillatory AFM experiments; however, mechanical modeling suggests that the nuclear lamina was not responsible for this strain-stiffening response.^59^ The characteristic relaxation time of nuclei has been shown to be dependent on the amount a nucleus has been compressed, as is consistent with a poroelastic material model.^68^ It is also important to note that the speed and duration dependencies and nuclear strain alter the nuclear mechanical response. AFM studies which reveal viscoelastic force response that increases with higher strain rate and monotonically decays as the strain is held fixed (viscoelastic relaxation).^64,240^ Users must be judicious in considering the rate of perturbation, as fast and slow deformation may selectively probe the viscous or elastic response of the nucleus, respectively. Finally, oscillatory perturbations have been recently shown to stiffen isolated nuclei,^60,98^ indicating a relationship between strain frequency and nuclear elasticity. Mechanical models of nuclei have been previously reviewed,^64^ but it is important to note that model choice can alter the conclusions of a study similarly to the use of the assay itself.

**FIG. 4.**
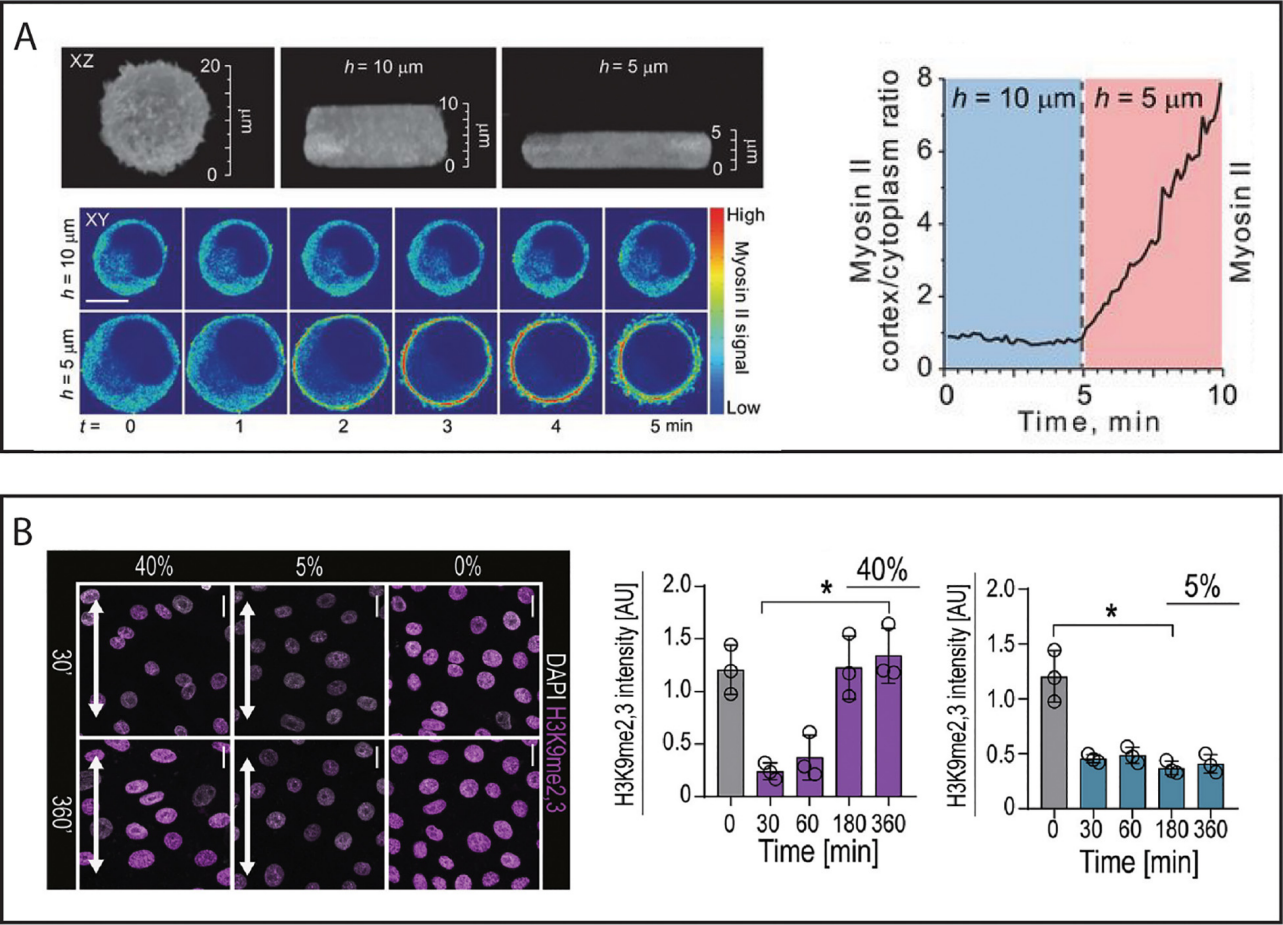
The importance of nuclear deformation parameters. (a) Lomakin *et al.* used parallel plate compression to study how nuclear confinement upregulates myosin activity. The increase in myosin activity was only observed for nuclear compression to a height of 5 *μ*m, and these myosin levels dynamically increase over the course of minutes. (b) Nava *et al.* used substrate strain to study force-induced changes in chromatin compaction. At 40% strain, chromatin decompacted after 30 min and steadily recovered over 6 h. At 5% strain, chromatin remained decompacted for the entirety of the experiment. All images are reproduced from their respective publications with permission. Images from (a) are reproduced with permission from Nava *et al.*, Cell **181**(4), 800–817 (2020). Copyright 2020 Elsevier. Images from (b) are reproduced from with permission Lomakin *et al.*, Science **370**(6514), 310 (2020). Copyright 2020 AAAS.

Strain magnitude, rate, frequency, and duration of an applied force have also been shown to affect biochemical readouts. Such dependencies are exemplified by two recent studies by Lomakin *et al.*^127^ and Nava *et al.*^13^ While studying the role of nuclear compression in myosin activity, Lomakin *et al.* discovered through a parallel plate compression assay that mechanically confining the nucleus induced an upregulation in myosin [Fig. 4(a)].^127^ However, the full story is more complicated. Not only does the myosin activity steadily increase over time following the mechanical confinement, but the height to which the nucleus was compressed dramatically changed the biological readout. Confinement to a height of 10 *μ*m saw no change in myosin activity despite corresponding roughly to a 50% strain. It took compressing a nucleus to a final height of 5 *μ*m for the subsequent change in myosin activity to occur. Therefore, altering the time post-compression at which myosin levels were measured and the final nuclear height subsequently would change the conclusions of the study. By sweeping of magnitude of strain and the observation time, the investigators were able to show existence of a strain threshold and dynamic changes in myosin levels. Though we have chosen here to highlight the results by Lomakin *et al.*, it is important to note that Venturini *et al.* simultaneously reported similar observations.^128^

Effects of strain magnitude and duration have also investigated in studies of nuclear extension. In examining how cyclic stretching of cell monolayers altered chromatin compaction levels, Nava *et al.*^13^ discovered a similar dependency upon both time and magnitude of the nuclear extension [Fig. 4(b)]. The investigators applied both 5% and 40% cyclic strain for up to 6 h, and observed the fluorescence intensity of H3K9me2,3 as a surrogate for chromatin compaction. Interestingly, they noted that at 40% strain, nuclei underwent chromatin decompaction within 30 min, which then steadily recovered to pre-strain values after 6 h. At 5% strain, however, nuclei reduced their chromatin compaction over the entirety of the 6 h of observation. Both responses were shown to reduce the strain propagated to the nucleus and subsequently reduce DNA damage. This study is a wonderful example of how the conclusions of a study could be jeopardized by not considering the deformation parameters. Had the investigators only observed nuclei 30 min-post strain, they would have simply concluded that cyclic strain led to chromatin decompaction. Similarly, if they only investigated 40% strain, they would not have discovered the separate mechanisms of minimizing nuclear strain and DNA damage. Similar considerations should be made for cell type and strain frequency in addition to strain magnitude and duration, as conclusions may be subject to these variables as well.^13,171–175^ These examples underline the critical importance examining the biochemical readout over a range of strain magnitudes and timescales when using physical perturbations to study nuclear mechanobiology.

## PERSPECTIVE AND OUTLOOK

The techniques and studies outlined here underline that understanding the details of nuclear mechanics, along with biochemical signaling, is a necessary piece in understanding biophysical mechanism. Most studies in nuclear mechanobiology thus far have focused on the ultimate biochemical response or structural remodeling resulting from mechanical stimuli. The next challenge is to fully map out the details of the mechanical mechanism between mechanical stimulus and biochemical response. Knowledge of how forces at the micrometer scale are distributed to molecular assemblies and how those structures are stretched, sheared, or compressed would improve our understanding of mechanochemical responses.

As with any collection of methodologies, each individual technique has a set of unique advantages and limitations that warrant or discourage its usage in various contexts. While it is important to focus efforts on improvements to individual methods that address their limitations, it is arguably more beneficial to adopt multiple techniques in studying a specific nuclear mechanobiological phenomenon. In doing so, one can appropriately overcome the weaknesses of a single technique to better establish a true mechanism or underlying feature. Take, for example, the work of Shah *et al.*^12^ In studying the role of nuclear compression in DNA damage induction, the investigators employed confined migration through both microfluidic devices and 3D collagen matrices, as well as leveraged parallel plate compression and AFM. This enabled them to compare (i) 2D vs 3D migration, (ii) migration-induced confinement vs physically induced confinement, and (iii) local vs global mechanical compression. Such comparisons improved confidence in the conclusions that nuclear deformation alone, independent of nuclear rupture, is sufficient to induce DNA damage in certain cell lines.^12^ The complementary use of several assays is not only relevant to studying downstream consequences of mechanical perturbation but also in studying the material properties of nuclei themselves.^241^ Comparative studies of commonly used assays have reported a wide discrepancy in measured material properties for the same cell type.^242^ It is, therefore, advisable to survey several techniques to better contextualize the material measurements. As the field progresses forward, it is the complementary use of several assays that will enable increased insight into nuclear mechanobiology.

Even when multiple assays to probe nuclear mechanical properties are used, the resulting output is commonly expressed as elasticity and/or viscosity that is meant to be indicative of the entire nucleus. The analyses and model choices used in the calculation of such material properties (e.g., the Hertz model for AFM), generally assume a linear, homogeneous, isotropic material under small deformation and at equilibrium. However, the nucleus is a highly heterogeneous,^242^ nonisotropic,^74^ strain stiffening^64,83^ nonequilibrium, active material. Furthermore, many of these models require the knowledge of the Poisson ratio, which is often unknown and could be cell line and/or condition specific. The invalidation of many of the underlying assumptions makes quantifying an elastic modulus or viscosity characteristic of the whole nucleus a gross simplification, and useful only for comparative measures and order-of-magnitude approximations. It is then prudent in our efforts to quantify material properties of the nucleus to move beyond this notion of global “nuclear stiffness” and progress toward measuring local viscoelasticity throughout the volume of the nucleus. The first step in such a process is to dynamically map displacement and strain distributions during physical perturbations, which has been practically realized in several instances.^100,101,206–208,243^ Further calculation of local material properties through mechanical models that do not *a priori* necessitate linearity, homogeneity, and isotropy could then provide maps of local material properties as opposed to the current standard of global measurements. Such analysis pipelines are active areas of research, and passive techniques such as image-based elastography^206^ and Brillouin microscopy^213,214^ have arguably provided the most headway. Understanding the spatial distribution of nuclear mechanical properties would be profoundly useful in understanding nuclear force transduction and how strain on the cell surface is nonrandomly propagated into specific genomic loci.

Many of the assays previously discussed exclusively deal with culture cells outside of their native environment. While studies of cell nuclei in this context are useful and have been foundational in establishing the field of nuclear mechanobiology, it is more relevant to perform these measurements *in vivo*. Such measurements introduce a variety of new challenges that are not present in studying cultured cells. First, many of the conventional microscopy methods that accompany the force measurement assays are severely compromised by deep tissue imaging. That is, refraction, scattering, and absorption lead to significant aberrations in the resulting images, thus making any quantification difficult or impossible.^244^ Advanced microscopy solutions such as adaptive optics,^245^ multi-view light-sheet fluorescence microscopy,^246,247^ and multi-photon microscopy^248^ provide means of mitigating such issues, but access to such equipment and the necessary imaging expertise can be a limiting factor.^249^ Furthermore, many of the methods describe previously, such as AFM and MM, simply cannot access individual nuclei *in vivo*. More broad-scale deformation techniques such as substrate stretching have been able to circumvent this,^250^ but there still exists a dearth of techniques capable of measuring nuclear mechanical properties within tissue samples. This motivates a new frontier of tool development research aimed at combining novel force probes with advanced microscopy to better enable studies of the physical role of the nucleus in tissue morphogenesis and embryogenesis.

## CONCLUSIONS

The past several decades have seen an exponential growth in studies on the physics of biological systems. More specifically, the subfield of nuclear mechanobiology has become an active area of research, garnering the attention of biologists, physicists, chemists, engineers, and microscopists alike. This is in part due to our understanding that the mechanical properties and morphology of cell nuclei are implicated in a myriad of rare and deadly diseases. Furthermore, it is also understood that the nucleus is a key player in confined migration in processes such as metastasis, which is responsible for a majority of cancer-related deaths.^251^ However, the mechanics of nuclei also facilitates healthy cell functions through transduction of mechanical forces into biochemical signals. The drive to quantitatively study such phenomena has unearthed a variety of new methodologies that span many scales from individual chromatin fibers to whole tissues. However, a keen understanding of the advantages and limitations of these assays is necessary. Here, we have provided a synopsis of the presently available techniques with further discussion of the specific biological questions for which they are well suited. Yet, there are numerous further considerations in selecting an assay, as we have described above. Specifically, one must first consider the tradeoffs inherent to increased specificity in strain application. The geometry of the force probe, as well as the length- and time-scales of the applied force, must be also be considered to properly interpret results. While the methods on hand have and will continue to provide useful insight into nuclear mechanobiology, further complementary use of multiple methods, studies of local material properties, and the development of assays for *in vivo* studies will continue to unravel the mechanical mechanisms that underlie these complex biological processes.

## Data Availability

Data sharing is not applicable to this article as no new data were created or analyzed in this study.
